# Modeling as Visioning: Exploring the Impact of Criminal Justice Reform on Health of Populations with Substance Use Disorders

**DOI:** 10.1177/23814683231202984

**Published:** 2023-10-11

**Authors:** Natasha K. Martin, Leo Beletsky, Benjamin P. Linas, Ahmed Bayoumi, Harold Pollack, Sarah Larney

**Affiliations:** Division of Infectious Diseases and Global Public Health, Department of Medicine, University of California, San Diego, CA, USA; Division of Infectious Diseases and Global Public Health, Department of Medicine, University of California, San Diego, CA, USA; School of Law, Bouvé College of Health Sciences, and Health in Justice Action Lab, Northeastern University, USA; Boston University School of Medicine, Boston, MA, USA; Institute of Health Policy, Management and Evaluation, University of Toronto, Toronto, ON, Canada; Crown Family School of Social Work, Policy, and Practice, University of Chicago, Chicago, IL, USA; Department of Family Medicine and Emergency Medicine, Université de Montréal, Montreal, QC, Canada

**Keywords:** modeling, decriminalization, opioid, drug, harm reduction

## Abstract

In the context of historic reckoning with the role of the criminal-legal system as a structural driver of health harms, there is mounting evidence that punitive drug policies have failed to prevent problematic drug use while fueling societal harms. In this explainer article, we discuss how simulation modeling provides a methodological framework to explore the potential outcomes (beneficial and harmful) of various drug policy alternatives, from incremental to radical. We discuss potential simulation modeling opportunities while calling for a more active role of simulation modeling in visioning and operationalizing transformative change.

**Highlights:**

- This article discusses opportunities for simulation modeling in projecting health and economic impacts (beneficial and harmful) of drug-related criminal justice reforms.
- We call on modelers to explore radical interventions to reduce drug-related harm and model grand alternative futures in addition to more probable scenarios, with a goal of opening up policy discourse to these options.

The overdose crisis has unfolded over the past 2 decades and surged during the COVID-19 pandemic in the United States.^1^ In the face of this crisis, many evidence-informed policy and clinical responses (e.g., overdose prevention centers, drug-checking services, safer opioid supply prescribing, reclassification of naloxone to permit over-the-counter sales in the United States, telemedicine solutions to prescribing medications for opioid use disorder) have demonstrated efficacy yet remain underutilized.

At the same time, criminal-legal responses to illicit drug use remain heavily resourced and disproportionately affect people of color—despite mounting evidence that the war on drugs has failed to prevent problematic drug use while fueling societal harms including disruption of community and familial bonds and exclusion from the formal labor market.^2^ While incarceration is associated with poor health outcomes generally, punitive drug policies and incarceration of people who use drugs are also associated with an elevated risk of drug overdose, HIV, hepatitis C virus, and tuberculosis.^3–5^

Frustrated by the failure of incrementalist reforms, social movements focused on racism, police violence, the carceral state, and drug policy are calling for a reenvisioning of how societies tackle these core challenges.^6^ Such reenvisaged drug policies include diversion and deflection interventions (programs that divert people with low-level criminal offenses away from the criminal justice system and into substance use disorder treatment and other community services but where drug possession remains illegal), discretionary policing (such as elective nonenforcement of certain criminal provisions to reduce the harm of drug markets), depenalization (dramatically reduced penalties or criminal-legal system attention on legal infractions related to personal drug use), decriminalization (removal of criminal penalties for possession of drugs for personal use, but where there is no structure to provide legal, regulated supply), outright legalization of particular substances (where the substance is permitted by law, generally implying a legal supply), prison abolition (reducing or eliminating the prison system and replacing it with rehabilitation systems and social welfare programs to reduce poverty and reshape structural determinants of health), and more. In practice, each of these policy changes could be written and enacted in different ways and therefore vary in both structure and impact.

A recent Lancet Commission on “Responding to the Opioid Crisis in North America and Beyond” recommended that “policies of incarcerating individuals for illicit possession of opioids or drug-related equipment intended for personal use should be abandoned because they present significant public health risks without offsetting public health or public safety gains.”^1^ Currently, more than 50 US counties and tribes are implementing diversion or deflection programs. Calls for decriminalization of drug use have led to policy change in some jurisdictions. The Netherlands, Czech Republic, Portugal, and Mexico, among other countries, have implemented various types of decriminalization reforms. In Canada, the province of British Columbia has begun a 3-y trial of decriminalization of personal possession of opioids, crack and powder cocaine, methamphetamine, and MDMA. In 2021, Oregon became the first US state to decriminalize drug use and expand access to addiction treatment and harm reduction services, More narrowly, nonmedical use of cannabis is legal in 23 US states and decriminalized in 8 states as of 2023.

Public safety challenges and their perception drive investments in policing and other carceral systems. Residents of low-income communities and communities of color report increasing concerns regarding crime and public safety. In many cases, such concerns are framed in terms that assume a link between policing and other security elements on the one hand and safety on the other. Proponents of strict sentences for drug-related crimes argue that the benefits offset the harms and that these policies deter drug use and associated crime. Although an effective police presence can deter crimes against persons, claims that public safety is enhanced though harsh criminal-legal sanctions focusing on drug use and sales are seldom confirmed.^7,8^ A complex debate about public safety policy is ongoing with special focus on benefits versus harms of continued investment in policing, prisons, and other elements of the carceral system.

Simulation modeling provides a methodological framework to explore the potential outcomes (beneficial and harmful) of various drug policy alternatives, from incremental to radical. However, the role of simulation modeling in operationalizing the health and health economic outcomes of this vision remains underexamined. The following commentary arose from a panel discussion presented November 10, 2021, at the Opioid Overdose Modeling for Policy Change Webinar that sought to explore these questions.

## The Role of Simulation Modeling

Epidemic and economic simulation modeling can aid policy makers in forecasting the population impact (both benefits and harms) and cost-effectiveness of different policy options. This is particularly useful when randomized trials are infeasible or difficult (e.g., decriminalization of drug use) or when trials are feasible but limited in their ability to track long-term outcomes (e.g., long-term impact on HIV or hepatitis C [HCV] transmission and mortality) and multiple outcomes (e.g., cost, crime, and health). Simulation models can provide a synthetic “test lab” to integrate data from multiple studies and estimate the complex and often interacting long-term health and economic impacts of policy changes. These simulation models can range in complexity from the relatively simple (decision tree or Markov models) to more sophisticated (compartmental, microsimulation, or individual-based network disease transmission models), depending on the question and data availability.

Modeling can be useful both before an intervention or policy change occurs (to assess theoretical potential impact) or after an intervention (to assess observed impact and project future long-term population impacts). Although there is currently only sparse and inconsistent data in select settings on effectiveness of more radical criminal justice reform policies (e.g., drug decriminalization or legalization) on justice involvement and health outcomes among substance using populations,^9,10^ there remains utility in using scenario modeling to explore potential policies and outcomes even prior to more widespread policy changes. As effectiveness data accumulate in settings exploring various types of drug decriminalization and diversion, modeling can be a critical tool in evaluating the current and future impact of these programs.

## Modeling Health Interventions in Criminal Justice Settings

To date, most models assessing the health impact of interventions in carceral settings for people who use drugs have studied incremental reforms and focused narrowly on impacts in one health domain, such as opioid overdose. For example, a modeling study in Rhode Island showed that medications for opioid use disorder (MOUD; i.e., methadone, burprenorphine, and extended-release naltrexone) at release from prison or jail would avert 5.8% of overdose deaths from 2017 to 2024.^11^ A follow-on study found that if MOUD was prescribed to all persons for whom it was clinically indicated in 2016, 1,840 deaths would have been prevented in the United States, with an additional 440 prevented if MOUD had been provided while they were incarcerated and postrelease.^12^ A modeling analysis in Australia showed that opiate agonist therapy (OAT, methadone and buprenorphine) provision reduced overdose and other-cause mortality among people who received it by 53% from 2001 to 2020 and that postincarceration OAT linkage accounted for 12% of the deaths prevented. Cost-effectiveness models of post-incarceration MOUD have generally focused on economic implications of reducing recidivism, but one evaluation of OAT upon prison release in Australia found it cost-effective in reducing mortality.^13^ In addition, a recent economic evaluation in Massachusetts found that providing all 3 MOUDs to incarcerated individuals and on release would prevent overdose and is more cost-effective compared with a naltrexone-only strategy.^14^ Importantly, community interventions can also have criminal justice impacts; one US study found that MOUD in the community reduces both health and criminal justice costs (through the impact of MOUD on reducing recidivism).^15^

Other models have focused on corrections-based treatment programs for infectious diseases associated with substance use disorder, such as HIV and HCV. These models examined the impact of HCV testing and treatment programs in prison in the United Kingdom, Canada, Australia, Ireland, and the United States, showing initiatives are cost-effective^16–20^ and can reduce HCV incidence in the community. Similarly, models have shown HIV testing and treatment in prison, jails, or on release is cost-effective in preventing HIV transmission the United States.^21–24^

## Modeling Foundational Criminal Justice Reforms

Numerous modeling studies have explored the potential impact of a highly localized form of drug decriminalization, enacted through overdose prevention centers (OPCs; also termed *supervised consumption sites*). OPCs are places where individuals can consume preobtained drugs monitored by staff who can intervene if an overdose occurs. Modeling studies based on Canadian data indicate OPCs are effective in preventing HIV, HCV, and overdose and are cost-effective in Canada.^25–29^ Theoretical modeling studies indicate OPCs could be effective and cost-effective in reducing HIV, HCV, overdose, skin and soft-tissue infections, and bacterial infections among people who use drugs in US settings.^30–33^

A handful of simulation modeling studiees have examined the impact of drug diversion programs and depenalization/decriminalization policy changes on health among substance using populations.^5^ Using observational data on a jail diversion program for low-level drug offenders in King County, Washington, modeling indicated this program could reduce HIV and HCV incidence by 3% over 10 y, reduce overdose deaths by 10% over 10 y, and was cost-effective.^34^ A theoretical analysis in Perry County, Kentucky, showed that a potential decriminalization reform, if resulting in halved incarceration/reincarceration rates and diversion to MOUD, could prevent more than half of new HCV infections among people who inject drugs (PWID) over 10 y.^35^ A study of Mexico’s 2012 public health–oriented drug law reforms, which depenalized drug possession and expanded diversion to drug treatment, used a modeling analysis based on longitudinal cohort data among PWID in Tijuana and found that a lack of implementation meant the reforms had little impact on HIV among PWID as of 2018. If fully implemented, however, these measures could prevent 21% of new HIV infections among PWID between 2018 and 2030.^10^

Few studies examine the economic implications of potential decriminalization coupled with reinvestment in public health approaches. A recent theoretical study showed that decriminalization in Belarus, Kazakhstan, Kyrgyzstan, and St Petersburg could be cost-saving (saving 38–773 million euros). Reinvestment of these savings into public health (HIV antiretroviral treatment and OAT) could prevent 59% to 84% of HIV infections among PWID over 20 y.^36^

Importantly, these analyses have explored a relatively narrow set of conceptualizations of “decriminalization” and associated benefits and harms. Further, as decriminalization includes multiple sectors (police, courts, carceral systems, health systems, social systems), so a systems-level approach to modeling is required to fully capture the implications across different sectors.

A movement for prison abolition has gained momentum, seeking to decarcerate (i.e., release currently incarcerated persons through review and reassessment of convictions and sentences), excarcerate (i.e., prevent incarceration through decriminalization of certain offences and strengthening social welfare and mental health systems), and develop alternatives to incarceration that focus on rehabilitation and restorative justice rather than punishment.^37^ To our knowledge, there has been no concerted effort to explore an abolitionist framework with simulation modeling. While a few modeling studies have examined the contribution of incarceration to health harms, where the hypothetical alternative is no incarceration, these studies do not explicitly explore abolition futures or frame these as policy options per se.^3,16,38^

## An Opportunity for Modeling Imagination

The above models demonstrate the potential benefits of incremental reform or narrowly visioned depenalization and decriminalization. These models are appealing because, like much drug policy research, they take a recognizable and current reality and build in change that may be viewed as politically and administratively realistic or actionable.^39^ One advantage of simulation modeling, however, is that we need not limit analyses to what is likely or even—in the moment—practical or feasible. Instead, models can facilitate speculation on alternative futures, producing results that serve to provoke discussion of a broad range of policy alternatives, even those that may seem unlikely or utopian.^39^ In doing so, models need not just enumerate outcomes of different scenarios; they can create and shape discourse around which scenarios are even possible, bringing alternative futures that may be seen as unrealistic into the realm of the achievable.^40^ Recognizing incarceration as just one policy option among many opens up opportunities to model futures that do not include carceral settings or radically reimagine their focus and remit and, in the process, create the potential for those futures to become reality.

What might such exploratory, speculative models look like? In the context of the overdose crisis, models exploring radical decarceration, excarceration, expungement, pardoning, reparations payments,^41^ resource shifting from law enforcement to mental health and substance use treatment systems, drug legalization, overdose prevention centers (which require exemption from federal drugs laws), and safer opioid supply prescribing may all be warranted. Different effects may be observed for decriminalization policies (thus affecting those who could potentially be incarcerated) compared with excarceration (thus affecting those who are currently incarcerated), and models can be used to examine these different populations. Outcomes could include health (overdose, HIV, HCV, skin and soft-tissue infections, mental health), drug use, crime, housing, criminal justice costs, economic productivity, considerations of health disparities and social equity, among others. Such models could compare outcomes to the status quo, providing both realistic enumerations of benefits and harms of alternatives and an explicit assessment of outcomes associated with current law enforcement–based responses to drug use.

Another advantage of simulation models is that they can facilitate the discussion of tradeoffs and potential unintended consequences. For example, abolition provokes understandable anxiety around the potential for crime to increase in response to specific interventions.^42^ Simulation models allow us to simultaneously examine health and crime implications of policy alternatives—and to examine contextual and program factors that may magnify or undermine a program’s intended social impact. Doing so is important, as policy makers likely will not take seriously an analysis that is unaware of such concerns. Data on drug offenses for the periods before and after decarceration efforts in response to the COVID-19 pandemic may be useful to this end.

The potential impact of this type of visionary modeling could be profound. We note historical and contemporary examples of academic researchers and other individuals afforded “authority” in policy making (e.g., physicians) in supporting grass roots harm reduction movements, including acts of civil disobedience such as the establishment of unsanctioned overdose prevention centers in the face of escalating overdose deaths and inaction from official channels.^43–45^ Simulation models provide a platform for generating the “what if” data that activists can use when speaking to policy makers and advocating for change. Furthermore, they can make explicit the hidden toll of status quo policies that goes unacknowledged in policy discussions because there is no counterfactual world to which to compare outcomes.

Together, incremental and more radical vision-changing models can complement each other in supporting policy making much in the same way that harm reduction policies have benefited from movements on each end of the spectrum. We therefore call on modelers to explore radical interventions to reduce drug-related harm and model grand alternative futures in addition to more probable scenarios, with a goal of opening up policy discourse to these options.^39,40^
